# That Prize Essay

**Published:** 1886-12

**Authors:** D. R. Wallace

**Affiliations:** Terrell, Texas


### BIBLIOGRAPHY.

### THAT PRIZE ESSAY!

Communicated for Daniel's Texas Medical Journal.

A PAPER of about 84 pages is published in the proceedings of the Texas State Medical Association as the essay to which was awarded the prize of one hundred dollars, offered by the Association for the best paper on a medical or scientific subject. The writer is J. R. Briggs, of Dallas. The committee of award were J. D. Osborn, of Cleburne, D. R. Wallace, of Terrell, H. K. Leake, S. D. Thruston and E. L. Thompson, of Dallas. There were eight or ten papers competing. To examine all these papers in Dallas during the session was simply out of the question. Time was asked for and granted. The whole matter was relegated to the committee—a mistake of which the Association, it is to be hoped, will not be guilty a second time. Some informal talk was had as to how we should proceed. It was at first proposed, as there were a majority of members at Dallas, if these agreed upon any essay, it should be adopted without reference to the other two. The proposition was obviously absurd. I did not object to it further than to state distinctly and emphatically that my name could be on no paper that I had not first examined. Whereupon it was suggested and agreed to, *nem con,* that the essays be kept in Dallas for examination one month—May—it was on the 1st of April when this agreement was made; on the 1st of June they were to be sent to me; I was to be permitted to retain them a month for examination, and then to forward them to the chairman of the committee; when, I naturally supposed, we would meet, organize the committee and discuss the merits of the *papers.*

Time was ample, the "Transactions" not making their appearance until November 15th, when I was permitted to see this paper for the first time. Of this I should not have complained had not the "Transactions" represented the whole committee as adopting the paper as the Prize Essay of the Texas State Medical Association. I should have sincerely rejoiced if, when I read the paper, I could have endorsed the action of the majority. True, I should have objected to having my name affixed to a paper as endorsing it, when, in fact, I had not been permitted to see it. A protest is the only thing now left me. I would have made a minority report in favor of some other paper, or of none, if none, in my judgement, were worthy; but I was not permitted to see the competing papers. My sole object in writing this protest is to set myself right before the profession and to do justice to the Texas State Medical Association, of which I have been a member from its organization. I am no stirrer up of strife, hate controversy, and I sincerely hope no offense will be taken at this protest. Whatever others may think of it, I regard the paper of Dr. Briggs as utterly worthless, and as not possessing a single qualification entitling it to be published in the proceedings or transactions. Here is the test:

"RESOLVED, That the several sections of this Association be requested, in the future, to refer no papers or reports to the Committee of Publication, except such as can be fairly classed under one of the following heads:

1st. Such as may contain and establish positively, new facts, modes of practice, or principles of real value.

2nd. Such as may contain the results of well devised original experimental researches.

3d. Such as present so complete a review of the facts on any particular subject as to enable the writer to deduce therefrom legitimate conclusions of importance." (*Vide Trans. for 1886, pp. 663*-47.)

These three rules constitute the *open sesame* to the door of the publication of papers and reports from the several sections. Most assuredly they should not be relaxed in favor of Prize Essays. What I have before said, I desire to repeat and to emphasize, I do not believe that the paper by Dr. J. R. Briggs, published in the "Transactions of the Texas State Medical Association," for 1886, has a single qualification entitling it to the honor of being admitted to publication, even, to say nothing of being awarded $100; to which I desire to add: *I believe I hazard nothing of truthfulness, and do injustice to nobody, when it is stated, as questionable, whether any paper on any subject, before any body of men, claiming to be working in the interest of science in any of its departments, ever found its way into its published proceedings, as worthless as the paper I, at present, refer to*, nor do I think the publication of this paper in the "Transactions of the Texas State Medical Association" will reflect any credit either upon the profession of the State, or upon Association, under whose imprimatur it goes forth to the public. These things I should prefer much not to have to say. I have been driven to it by a sense of duty to myself, and to the Association. I wrote two letters to the chairman of the committe to place me right before the public. I take the liberty to quote his last letter, as showing his animus: "I have," he says, "no minority report to make," alluding to my expressed wish to be set right before the profession, and a few lines after, he says, (showing he does not regard the Prize Essay as an epoch-making contribution to medical literature) "very few of the Texas State Medical Association will ever *wade* thro' the nonsensical (to me) article." Thus, it seems, Dr. J. D. Osborne consoles himself. I *know* many eyes in, and not a few out of Texas, will glance over these pro ceedings, and the *Prize Essay* will attract especial attention, and go far in forming an ideal of the profession in Texas. My name stands for something, in as out of the profession in Texas—*not much—how little*, no one is more conscious than myself; but whatever it is, *it is the result of work from the ground up*, and I do not propose to be elbowed into prostituting it. I care not what others think, it is their right. What I do care for is, that I should be made to say what I think false—placed in a false attitude to my professional brethren, and made to betray the interest, so far as my name stands for anything, of an association I love with every fibre of my being. The Publishing Committee decided not to publish it, and appealed to the President. He decided, very properly under the circumstances, to publish it.

It is noted, with regret, that DANIEL'S TEXAS MEDICAL JOURNAL announces that I will review Dr. Briggs' paper in the next issue of that journal. I could not think of such a thing; he would be a brave man who could. To an audacity of genuis of which few are possessed, he would need to be endowed with a mental acumen and inspirational insight to descry airy, dreamy nothings at distances most wondrous, as I think I will show you when, I come to point out a few of the beauties that are to be found in this *Prize* Essay. Indeed, I know of nothing like them in all literature. Francois Rabelais, whom Lord Bacon styles the "Great Jester of France," and others, the "Comic Homer," uniting in his single person, doctor, priest and author, might have been equal to the task. The incomparable genius displayed in Gargantua and Pentagruel show he would have been the man to grapple with such a peerless production. But born in 1490, he has long since become historic. In the plaintive words of Mark Twain, when speaking of one of Rabelais' cotemporaries, who was the architect of the great Cathedral of Milan,—"but he is dead now,"; so that the *Prize* Essay of the Texas State Medical Association will have to drag its slow length along down to posterity, deprived of critic capable of explaining its wonderful knowledge, and scientific explorations, to the admiring gaze of the millions yet unborn. The writer of the Prize Essay is known to me, so far as known at all, a reputable medical gentleman, and one of the associate editors of the Texas Courier-Record of Medicine. The gentlemen who made the award are honored members of an honorable profession. I have the profoundest respect for the personal character and professional status of all of them.

"How came Dr. Briggs to write so poor a paper as you characterize the Prize Essay as being?" Will the reader have the goodness to indulge me in attending to one more little matter, and I pledge to tell him, aye, to show precisely why he did not write a better paper. Let us first, however, settle another question; how did such a paper find its way through such a committee to the unfortunate place (so unfortunate to nobody as to the writer) which it holds in the annals of the State Medical Association of Texas? This is precisely the question that has given me the headache for two weeks, trying to solve it. Of one thing, and one thing only, am I perfectly sure, and in all my guesses I begin by taking it for granted, viz: whatever may turn out to be the true solution, these gentlemen are men of the first water, as eminent in their social relations as in their professional status, who would not knowingly do wrong. Further than this we dare not go, the whole being a mystery as securely guarded as the frozen secrets of the North pole. The writer has puzzled himself again and again over this very question. He has ransacked his small store of reading from nursery tales and wild western scenes up, and on, through Humboldt, and Darwin, and Spencer, and Tyndall, and Huxley, "*et id omne genus*," to find something like it–all without result. With aching head, and corrugated brow, I was startled by one of those strange dim reminiscences carrying me back over the waste of long ago. I stop, turn my eyes up—Eureka! I have it; I am sure of it. By the blessed Virgin and all the Saints in the Calendar! if not like, it is a parallel! It is an incident recorded in Knickerbocker's History of New York, by that genial soul of blessed memory, Washington Irving, with whose veracious Chronicles our school-boy reading must have made us all familiar. I am sorry its insertion here requires so much space, but it is so invaluable in conducting to what may enable us to clear up this great mystery, before the mould and dust of time settle, as you know they will, on great events like this; but I feel assured I will have your thanks.

The incident referred to is connected with the beginning of the administration of the redoubtable Wonter van Twiller as governor of the Nieuw-Nederlandts, his capital being New Amsterdam, now New York City.

"The very outset of the career of this excellent magistrate was distinguished by an example of legal acumen that gave flattering presage of a wise and equitable administration. The morning after he had been solemnly installed in office, and at the moment that he was making his breakfast from a prodigious earthen dish, filled with milk and Indian pudding, he was suddenly interrupted by the appearance of one Wandle Schoonhoven, a very important old burgher of New Amsterdam, who complained bitterly of one Barent Bleecker, inasmuch as he fraudulently refused to come to a settlement of accounts, seeing that there was a heavy balance in favor of the said Wandle. Governor Van Twiller, as I have already observed, was a man of few words; he was likewise a mortal enemy to multiplying writings, or being disturbed at his breakfast. Having listened attentively to the statement of Wandle Schoonhoven, giving an occasional grunt, as he shovelled a spoonful of Indian pudding into his mouth, either as a sign that he relished the dish, or comprehended the story—he called unto him his constable, and pulling out of his breeches pocket a huge jack-knife, dispatched it after the defendant as a summons, accompanied by his tobacco box as a warrant.

This summary process was as effectual in those simple days as was the seal ring of the great Haroun Alraschid among the true believers. The two parties being confronted before him, each produced a book of accounts written in a language and character that would have puzzled any but a High Dutch commentator or a learned decipherer of Egyptian obelisks, to understand. The sage Wonter took them one after the other, and having poised them in his hands and attentively counted over the number of leaves, fell straightway into a very great doubt, and smoked for half an hour without saying a word; at length, laying his finger beside his nose, and shutting his eyes for a moment, with the air of a man who had just caught a subtle idea by the tail, he slowly took his pipe from his mouth, puffed forth a column of tobacco smoke, and with marvellous gravity and solemnity pronounced that, having carefully counted over the leaves and weighed the books, it was found that one was just as thick and as heavy as the other—therefore it was the final opinion of the court that the accounts were equally balanced—therefore Wandle should give Barent a receipt, and Barent should give Wandle a receipt, and the constable should pay the costs. This decision being straightway made known, diffused general joy throughout New Amsterdam, for the public immediately perceived that they had a very wise and equitable magistrate to rule over them. But its happiest effect was, that not another lawsuit took place throughout the whole of his administration, and the office of constable fell into such decay that there was not one of those local scouts known in the province for many years."

Now may this not be a pointer? it may not, aye, it could not have been just after this fashion, but something like it. For instance, the three committeemen met—they are all busy men—do a large practice—have a numerous clientelle. They survey the huge pile of munuscript—it looks formidable. The one that is to be (so the fates have ordained) prize essay, looks portentously voluminous—it is a whole book; it is written with a type-writer. Still, its aspect is forbidding to the busy practitioner, as all these gentlemen are. They *eye it*; it is on a psychological subject. The busy practitioner of general medicine has little fancy for such reading. They compare it with the others. It must have required a great deal of valuable time to get it up. There are 84 printed pages, 2,436 lines and 24,360 words. Now, they are run for time—have a time-honored precedent in the Wonter Van Twiller decision, that, so far as known to history, has never been questioned. "Why not?" suggests one, "settle the matter *a la mode* Wonter Van Twiller?" "It is a good suggestion," responded one of the others, who had half a dozen calls awaiting him. It is agreed—they proceed methodically—they count pages, lines, words, &c.; estimate them, and find Dr. Briggs' paper to distance all competition by several thousand words. Withal a very clever gentleman, as he lives here with us, we know him to be a clever fellow. No doubt he deserves it, as his manuscript is much heavier than any other. *Jacta est alea*—the die is cast, and Dr. Briggs is declared victorious! It is not stated that this was really the mode resorted to. It is but a guess. In the absence of all light upon the subject, it is permitted to guess. It is true, I was one of the committee, but I was not permitted to be present, but I am certainly permitted, without offense, to guess at a mode of procedure at which I had a right to be present.

This troublesome matter disposed of, the writer proceeds to show the reader a few specimens of this wonderful production, and how it was, its author did not do better.

Let us glance, in introducing the subject, at the Doctor's style (if he may be said to have any). It is not to be inferred that he has not words, as he has them in great abundance. Yet his cannot be classed as a *redundant* style. Blair's is a redundant style, but it is also very clear and easy to read—the words are drawn out, &c. It is not an *obscure style*, although one seldom knows what he is talking about. The more one looks at and studies over an obscure sentence of a great writer, the more the sense stands out. But take one of Dr. Briggs' obscure sentences and, I know not how it may be with others, but to me, the more I study, the less I see in it, the worse confounded becomes the confusion. Then the style is not an obscure one; certainly it cannot be classed as ornate, for though he uses sesquipedalin in abundance, the whole cannot be classed by rhetoricians as ornamental. The Germans have a word they sometimes apply to style, that seems to me just describes the Doctor's method of putting ink on paper—a word, it is hoped, that will be the less objectionable to the Doctor, being of "learned length and thundering sound"—a jaw-breaker and throat dilator; it is *zusammengeschneidert*—meaning a style in which words are arranged on paper, after the fashion of a sort of patch-work, in which bits of cloth of different sizes, color and fabric are sewed together at random—a sort of *crazy quilt gone mad.* It occurred to me, in trying to classify the Doctor's style, that this comes nearer to it than any I could recall, in running over in my mind the small amount of rhetoric I can now recall—(more than thirty years have elapsed since I looked into a treatise on the subject.) If I have failed to assign him to his proper classification, I beg pardon of the reader, and the Doctor, more especially.

We now proceed to quote some specimens of it, and let the reader judge for himself.

In the first paragraph he says: "It seems to me we, as physicians, are too prone to grasp *gross facts* rather than turn the channels of our investigations into the rich fields of forces and substantial, though invisible entities which broad domain, with its protean manifestations are, on every side, legion." Just what the writer intended to mean by this short paragraph of four lines, I am sure I do not undertake to say; but I gather he deprecates as comparatively worthless, "*gross facts*," and invites us to turn the channels of our investigation into what he terms the *rich field of forces.* Perhaps the good Doctor will be so kind as to tell us what *gross facts* he alludes to, and where the "rich fields of forces," unobstructed by the "gross facts," are; and where dwell those "invisible entities" that constitute that "broad domain" which "are [is] legion"?

I was under the impression that phenomena, (not "noumena") facts, all indications and manifestations of matter and mind, were what concerned the scientist; that the whole catalogue of essences, quidities, entities and whatnot constituting ontology had been relegated to the owls and bats; for, says Maudsley: "After being in fashion over two thousand years, nothing has been established," by these ontological pursuits; and Lord Bacon says, in regard to all such discussion, "not only what was asserted once, is asserted still, but what was a question, is a question still"; and instead of being resolved by discussion, is only fixed and feed. For instance, it was observed that water would rise to a certain height in a vacuum; here was a *gross fact.* The doctor's sort of scientist immediately exclaims in the language of his ontological or metaphysical trumpery. "Behold, nature abhors a vacuum"; "no," replied one of these hard-headed *gross fact* loving fellows, "no sir, the height of the water is an exact measure of the weight of the atmosphere"; another *gross fact,* more general.

But to proceed; he begins his second paragraph with these words: "Here is an inexorable quality of physical and mental law." What, doctor? You have told us of no such law, and you tell us further, "it is not an abstract, theoretical myth, but a veritable *vascular force.*" Now you *have* us! We thought it was abstract things you were looking for, not *gross facts.* Have the goodness to tell us what *vascular force* you mean, and what it has to do with your subject? In the next sentence you say, "*this vital life force*" does not cease with moulding the body, but gives to our intellectual and mental qualities, shaping, as it certainly does, our character through life," &c. What a vital force is, we know or have some idea of; we also know what a life force is, but what are they when blended? But this, by the way, what is it that is given? What is the object of "gives?" What is it that is given "to our moral and intellectual qualities?" As the grammarian would ask, what is the object of the transitive verb "gives?" "It shall be our purpose to show the mental forming element in *hereditary transmission* of diseased action, whether of a physical, mental or moral character." Minerva defend us! What and where is the mental forming element in disease, hereditary or not; and where, in the Essay, is it pointed out? But my space is becoming beautifully less—I must hasten to page 546: "It is indeed surprising to find so little written upon rational principles pertaining to results accruing from hereditary predispositions beyond that of bare clinical facts, which, if unformulated, are utterly worthless to the practitioner of medicine. In fact, little is said in any way respecting hereditary disease—our standard text books devoting casual, indefinite attention to this subject." On page 549, "In this elucidation we rely mainly upon our own practical observation, for we are forced thus on self-reliance, owing mainly to the scarcity of literature for reference." On page 550: "While it is an actual fact, but little has ever been said or written upon the laws of hereditary influence, it is one of the most vital questions with which the modern physician should have to deal." And this by one writing a competing article for a Prize Essay, and actually obtaining it! Whether ignorance or presumption has the better in these lines is left to others to settle. The writer has been a reader of current medical literature for 35 years; he has seen nothing like it, nothing second to it. For one to be uninformed upon some fact or principle in any department of medical literature is matter of surprise to nobody; but for one as perfectly ignorant of the whole subject to make such a statement as is contained in the above excerpts, to think, because *he* knows nothing of the literature of a subject none exists–well, the reader may name it. Of just the sort of literature of which he complains of a dearth, there has been quite a disproportionate quantity. There has been, and continues to be, since about 1860, or about the beginning of the publication of Mr. Darwin's labors, an interest in these subjects never previously manifested. I cannot quote them all, as I would have to make an inventory of my library. I mention a few writers: W. B. Carpenter's Physiology; Mental Physiology, by same author; Physiology and Pathology of Mind, by H. Mandsley; Body and Mind, by same author; *Influence of the Mind Upon the Body*, (large vol.); Insanity and its Prevention, both by Dick Hark Tuke; Psycological Medicine, by Bucknell & Tuke; Frerich, 2 vols., first especially; Echeverria on Epilepsy; Insanity and its Treatment, by Blanford; Brain as an Organ of Mind, by Bastian; Pepper's System of Medicine, vol. v; *Action ond Re-action of Mind on Body*, in Tuke's Influence of Mind on Body; Treatise on Insanity, by W. A. Hammond; The Journal of Insanity, published by the late J. P. Gray for the last 30 years. The yearly Reports of Superintendents of Asylums in England and the United States and Canada, in numbers of which the subject is handled with consummate ability. These are mentioned as being the more common sources of information on the subject. Hundreds of others less known might be mentioned, which being less common, it is thought less fair to mention. Works of Schroeder, Van Kauk, and Mayer, of Germany; of Matet, of France; Tamburini, of Italy; Theodore Meynert, of Vienna, are too important to omit. With the labors of these, and such as these, it seems Dr. Briggs has no acquaintance. Then from this *coigne d' advantage* I am prepared to answer, at least in part, why the Doctor did not write a better paper than he is represented to have done. *He knew nothing of the subject.* If he had read one page in a thousand he would not have written on the subject at all, or if he had, he would have made himself acquainted wiih the literature of the subject, about which he knows no more than the busy practitioner has time for; it would have taken him some years. I take it, when one takes in hand to write a Prize Essay, he owes it to himself, as well as to his readers, to make himself acquainted, first of all, with the literature. According to his own confession, Dr. Briggs did not do this, and as proof of it, throughout an 84-page Essay he has not quoted a single authority, a thing, so far as my information extends, unprecedented in the annals of science. Dr. Briggs may be a very great man—that, at least, is not in question, but it is not in the limits of the possible for one man's head, however capacious, to hold everything. Another disadvantage, under which he evidently labors, is his avowed aversion to GROSS FACTS. A greater than he can ever hope to be said, ',He that builds without a *material* basis builds a castle in the air."

To return to the quotation: What is meant by "so little written on rational principles?" What is written—and hundreds of thousands of pages have been written—is in accordance with the best, highest and broadest principles known to the greatest intellects working in the profession. What is meant by "bare clinical facts unformulated?" I know of no such facts. As fast as discovered and fairly established, every fact in medicine is seized upon, pressed into science, and made to do service, in helping the great science onward and upward to higher generalizations and broader views. Has the Doctor found some means in the use of which he can make *gross facts* (gross is his word; I know no difference; the facts of science are simply facts, to the scientist) yield better results than they have been doing? Then why not give the profession the benefit of his modus? After the first few pages he deals in nothing but platitudes of what is wrong in society, not a thing that any well informed citizen does not know just as well as he. It is true, he speaks of them in a sort of magisterial *ex cathedra* way, as though his thoughts were emanations from the fountain of light; but if he mentioned one evil that exists in society, that is not common property, or one measure of reform that is not mentioned in the books, even in current newspaper literature, I trust he will have the kindness to point it out.

But to return—In this age of utilitarianism every fact in every department is seized upon and made to do service in pushing forward the car of progress. It is to be hoped we shall hear no more about gross unformulated facts.

"When brought," the Doctor says, "to a calm consideration of the question, we are at once convinced that many diseases with which we are daily contending are the legitimate results of inheritance." Now, Doctor, do you have to be brought to "a calm consideration" before you know this? If so, you are the only doctor that I ever knew who had to prepare himself in any way to be convinced of it. We see it every day in practice; you have just said, "In fact, but little is said in any way about hereditary diseases, our standard text books, merely devoting casual and indefinite attention to the subject." Now, my dear Doctor, while you did not recognize the fact that heredity cut as much figure in disease as you have lately discovered it does, do not conclude that other medical men's eyes were shut to this patent fact; and in similar wise because you are not acquainted with the literature of the subject, I pray you, not to conclude none exists; I assure you it exists, cartloads of it; a very little of which, had you acquainted yourself with, you would not have written the essay under consideration.

Page 547. "There have been two and distinct classes of thinkers upon the subject of man's *environment* from the first days of human records, one purely and solely materialistic in character, advocating the evolution of man from the lowest forms of inferior animals; the other, enthusiastic expounders of a highly spiritualistic theory of man's transcendent height above everything else of an animal nature."

This question will be continued, but it is thought best to stop here and ask: What is meant by the subject of *man's environment?* It is taken for granted, the writer means *origin,* as the words following would make no sense whatever with *environment.* The sense, as I understand it, of the period is: There are two theories of man's origin; one, according to Genesis, that he was made by the fiat of God, out of the dust of the earth; the other, that he is the result of his environment, the forces inherent in nature produced him. So far, so good. We now have a third theory of man's origin by J. R. Briggs, M. D., author of Prize Essay of the Texas State Medical Association for 1886. Here it is: "A middle ground will, however, show the incorrectness of either view, when sifted down to the actual truth." Hide your diminished heads, ye venerable Christian sages! Paul, Origen, Augustine, Anselm, Luther, Calvin, Cramner, Latimer, Knox, Spurgeon and Talmage, and you too, ye Goethe, La Place, St. Hillair, Darwin, Spencer, and all the balance of you; your faith avails you, on the one hand, no more than your science on the other; the one (the scientific) is "cold and heartless;" the other (scriptural) is "rampant with ill directed courage." Here is the solution of the origin of man that the fools of faith and the votaries of science have been seeking in vain,—"Clinical experience plainly developes the absurdity of the Atheistic teaching, while it also shows that man is anything but an angel, and therefore the doctrine of evolution and abstract metaphysics can never agree upon such diverse views." You are expecting something else of this man-origin theory? No, sir, there is no more; just *four lines* did the business. "But it is not clear what it is, after all," says somebody. I reply, it is as clear as *mud.* "It is the absurdity of Atheistic teaching" combined with "man is anything but an angel" that is the origin of man!!! He told us the others were no good, and man *must have* an origin; and having told us what it is not, he is not going certainly to leave us without a God; so it must be, what I have stated it is, or this—"and therefore the doctrine of evolution and abstract metaphysics can never agree upon such diverse views." It may be this latter,—it being so important to the man or woman of common intelligence to be informed *that evolution and metaphysics "can never agree in such diverse views."* What "diverse views" are here alluded to, it is not very clear; but it is not reasonable to be too exacting with one who has just had the goodness to unfold to us the origin of man. Better be patient and wait for another revelation.

Page 549. "It occurs to me as a remarkably strange fact that no advantage whatever has been taken of inherited, inborn and acquired predisposition for the improvement of the race of man." Strong language this; Dr. Briggs must have come by his knowledge of the subject very lately. Not a man of a family ot ordinary intelligence in the land, but knows just about as much of the matter as does the Doctor or myself, and practices it, in marrying himself —in marrying off his family—in selecting a profession for his sons; one son is taken from school—a clerk's desk or the profession of a teacher, or of a minister, even against his inclination, not seldom against his will; boys and girls are made to exercise in the open air; all these things for no other reason than *"inherited, inborn and acquired predisposition."* It is matter of wonder, the learned writer never heard of this thing. He says so. We must believe him. He says emphatically "no advantage whatever has been taken of it"; so it follows, of course, he never heard of any. But, take my word for it; you have been as ignorant of what has been going on around you, as you are of the literature of the same subject. In the next line to the one quoted above, the words "these holy and magnanimous laws" occur. Now it may occur to others as to me, I see nothing holy or magnanimous in these laws. Like all others with which we have been endowed by the Creator, they may be said to be holy but hardly *magnanimous,* which, compounded of two Latin words, *animus* (spirit) *magnus,* (large, great), must mean if anything, high spirited, great spirited, large spirited; hardly a description to apply to a law *"inexorable* and *unconscious."*

Page 551. "Being guided by our intellectual and moral qualities in all the ways of life, it would seem rational that we seek to better the human race, by developing our intellectual conscious nature." I have never noticed what is here taken for granted, as a well known fact, that "we" (doctors) are "guided by our intellectual and moral qualities in all the ways of life" any more than other people, and when we are, I make no question, we will do as the Doctor thinks we ought, in the direction indicated.

I am about through with a painful duty; how painful, few can realize. It is true, I have called attention to a few things only, on the ten pages, as a specimen of the prize essay. There are hundreds of others just as objectionable to good sense, taste, and, in fact, all the qualities that go to make up a common sense readable essay, such as this aspires to be. The other seventy-four pages are of about a whatness—dull, dead, poorly expressed platitudes of what everybody knows in, and out of the profession, set forth with a flourish, as of oracles. Ever and anon, he goes a step or two from the beaten track, only to be caught and entangled in the brushwood of the unknown region, into which he imprudently ventured, and from which, he hastens back to the safe highway. What Carns, in his controversy with Hahnemann, as quoted by Bartholow, says, of homœopathy, is equally true of the prize essay: "Whatever is new is not true, and what is true is not new."

I conclude with some excerpts just by way of giving specimens of the style, beginning where the book happens to be open before me. "While the *questions* (I italicize the beauties in the turn of expression and vigor of thought) may not have the scientific ring usually sounded by the physicist and biologist of modern times"; (they certainly have not) "yet they are, *however*, of great moment (to Dr. Briggs and no one else) and *must be looked to*, if we would be scientific in all the term implies." (Why certainly,) "The scientific physician is the guardian of the human race." (In the name of all the gods at once, upon what meat do we doctors feed that we have all at once become so great?) "Men and things are his, and upon his acquirements and prompt action depends the character of all subsequent physical and mental states"!!! I had no idea I belonged to so royal a profession. Fool that I have been, not to have realized my position sooner; but the fact is, I have gone through life, and become an old man, feeling I was no better than other people. Why, our grandams would not know us. "However, more will be said upon this part of our subject further on. Suffice it to say, that *we must, with the present lights before us*, hamper and paralyze our will power and conscience, if we stand quietly by *with stoical indifference*, in the midst of such profound results." What, good doctor, is going to happen to us, if we let people attend to their own business? (Oh! this terrible paralysis of conscience and will power!) "These reflections merely show the importance of our subject; for without this feeling of deep interest, we will not be calculated, with all our boasted learning to do more than retrograde to the primative haunts of the barbarous." (Do, Doctor, save us from the *primative haunts*; who has boasted of learning but yourself? It is to your strong arm, and learned head we turn for help.) "Doubtless some will say, that medical science has only to do with the tangible. This being true, then we find ourselves literally bewildered with a myriad of forces around, in, and through us, which can only puzzle and confuse." (Doctor, this is true of us common mortals. We do know nothing of these forces, only as we see them manifested in matter, ("tangible") but not so you, Doctor, you need not "puzzle and confuse" yourself about matter and *gross facts*, but just lay hold of the pure forces and put them in harness, and make them work for human-kind, and keep us all from those "haunts of the barbarous."

"Now it seems to me that a logical *production* based upon a clinical experience relative to the action or law existing between the mind and body, is a step in the proper direction!!!!" (What a profound discovery and how beautifully expressed. Oh! if Carpenter, Maudsley, Tuke, Bain and Hamilton had had this profound "logical production" before them when they were writing their great works on this subject!!) "Yellow fever and cholera will scare us into the most immediate and enthusiastic wildness." (Oh! if I could see one of these fellows in a state of *most immediate and enthusiastic wildness!* Now the description of the fellow described in the next sentence, after the one quoted, "contracting syphilis and injecting the life-long suffering death-warrant into the unborn innocents." (I know about how these fellows look in these operations.) "And so it is to right ourselves, by pointing *out our certain disturbances, now affecting us*, that we call special attention. The economy of physical and mental culture lies in the discovery of all abnormal disturbances. These disturbances are now pointed out and means proposed for their obliteration." The God be blessed! We are now on the highway to the Millennium!

On page 570 the writer of the Prize Essay gives us a most edifying bit of autobiography, in which he places the world under an immense debt to him, by relating of what he was conscious, "*while I was utterly unconscious.*" (I cannot be mistaken; these are his words. He says he was utterly unconscious and comatose four days and nights, and yet he has the goodness to tell us of what he "was conscious" while "utterly unconscious.")

But reader, for your sake I stop; there are hundreds of more things that might afford matter of merriment, but this will suffice to show what that Prize Essay is.

The circumstances, not the value of the Essay, (it might have passed into its merited oblivion) have dignified it with this notice. I have written without the least pains-taking, interrupted every few minutes. As a public document belonging to the Texas State Medical Association, I have simply exercised a right I had to express my opinion on this remarkable production; in doing so "nothing have I extenuated, naught set down in malice." For the writer I have cause to entertain nothing other than the kindliest feeling. For the Dallas members of the committee I hold them, as they are generally held in the community in which they live, as polished ornaments of society and as professional gentlemen.

Before signing my name to this communication, I have to make this proposition to Dr. Briggs: Submit his paper to three recognized psycologists, (I care not who,) say Hammond; Seguin, of New York; Parrish, of Burlington, N. J.; Everts, of Cincinnati; Jewell, of Chicago;—others equally qualified can be chosen—he to select one, the President of the Association another, and the third by myself. If they decide his a Prize Essay, I will pay him $100 additional; if not, he returns the $100 prize money to the treasury of the Texas State Medical Association.

D. R. WALLACE, M. D.

Terrell, Texas, Dec. 10, 1886.

---

OFFICE DANIEL'S TEXAS MEDICAL JOURNAL,
December 20, 1886.

DEAR SUBSCRIBER:—"Christmas comes but once a year.'. It is here now. If you have more money than you know what to do with we would suggest that *we* have not, and that a division, at least to the amount of one subscription, would be an acceptable Christmas gift. This is the season for renewal of subscriptions, and there *could not be* a more appropriate time. We continue to accept postal notes, post-office money orders and bank drafts; and, if *we must*, (as a special favor,) we will accept a limited amount in currency, or even silver. Don't all speak at once. Yours truly,

D.' T. M. J.

