# Errata: Dr. Wallace’s Review of the Prize Essay

**Published:** 1887-01

**Authors:** 


DR. WALLACE'S REVIEW of the "Prize Essay" T. S. M. A. which appeared in the last number of the JOURNAL contained some errors, typographical and clerical, to which we wish to call attention here. "Wonter Van Twiller," read" Wouter Van Twiller" page 247 and 258; for "sesquipedalin," read "sesquipedalia," page 249; for Dick Hark Tuke" read "Dick Hack Tuke"; for Van Kauk," read "Vander Kolk," page 252; for "Carns," read "Carus," page 256; and on page 254 seventh line from the bottom, for "God," read "origin."

